# Respiratory modulation of oscillometric cuff pressure pulses and Korotkoff sounds during clinical blood pressure measurement in healthy adults

**DOI:** 10.1186/s12938-016-0169-y

**Published:** 2016-05-10

**Authors:** Diliang Chen, Fei Chen, Alan Murray, Dingchang Zheng

**Affiliations:** Department of Electrical and Electronic Engineering, Southern University of Science and Technology, Xueyuan Road 1088#, Xili, Nanshan District, Shenzhen, China; School of Electrical and Electronic Engineering and Faculty of Medical Sciences, Newcastle University, Newcastle upon Tyne, NE1 7RU UK; Health and Wellbeing Academy, Faculty of Medical Science, Anglia Ruskin University, Chelmsford, CM1 1SQ UK

**Keywords:** Respiratory modulation, Oscillometric cuff pressure pulse, Korotkoff sound, Blood pressure measurement

## Abstract

**Background:**

Accurate blood pressure (BP) measurement depends on the reliability of oscillometric cuff pressure pulses (OscP) and Korotkoff sounds (KorS) for automated oscillometric and manual techniques. It has been widely accepted that respiration is one of the main factors affecting BP measurement. However, little is known about how respiration affects the signals from which BP measurement is obtained. The aim was to quantify the modulation effect of respiration on oscillometric pulses and KorS during clinical BP measurement.

**Methods:**

Systolic and diastolic BPs were measured manually from 40 healthy subjects (from 23 to 65 years old) under normal and regular deep breathing. The following signals were digitally recorded during linear cuff deflation: chest motion from a magnetometer to obtain reference respiration, cuff pressure from an electronic pressure sensor to derive OscP, and KorS from a digital stethoscope. The effects of respiration on both OscP and KorS were determined from changes in their amplitude associated with respiration between systole and diastole. These changes were normalized to the mean signal amplitude of OscP and KorS to derive the respiratory modulation depth. Reference respiration frequency, and the frequencies derived from the amplitude modulation of OscP and KorS were also calculated and compared.

**Results:**

Respiratory modulation depth was 14 and 40 % for OscP and KorS respectively under normal breathing condition, with significant increases (both *p* < 0.05) to 16 and 49 % with deeper breathing. There was no statistically significant difference between the reference respiration frequency and those derived from the oscillometric and Korotkoff signals (both *p* > 0.05) during deep breathing, and for the oscillometric signal during normal breathing (*p* > 0.05).

**Conclusions:**

Our study confirmed and quantified the respiratory modulation effect on the oscillometric pulses and KorS during clinical BP measurement, with increased modulation depth under regular deeper breathing.

## Background

Manual auscultatory and automated oscillometric methods are non-invasive ways usually used for blood pressures (BP) measurement. The manual auscultatory method is based on the auscultation of Korotkoff sound (KorS), while the automated oscillometric method is based on the analysis of oscillometric cuff pressure pulses (OscP). The reliability of OscP and KorS signals are therefore important for accurate BP measurement for both manual and automated methods. It has been widely accepted that respiration is one of the main factors affecting BP measurement [1]. Published studies have shown that respiration influences both systolic and diastolic blood pressures (SBP and DBP) [1–5]. With regular deep breathing, both SBP and DBP decreased significantly by 4.4 and 4.8 mmHg respectively, in comparison with normal breathing [6]. According to a major review in the Journal of the American Medical Association (JAMA), a systematic error of 5 mmHg error of BP measurement would result in 27 million Americans being exposed to unnecessary treatment or 21 million being denied treatment [7]. Therefore, it is clinically important to understand the effect of respiration on OscP and KorS.

Until now, the underlying mechanisms of the effect of respiration on OscP and KorS and on BP measurement have not been fully understood. It is generally believed that respiration influences the central venous pressure through the chest expansion and compression. Inspiration decreases central venous pressure, leading to the increase of venous return and right atrial filling and the decrease of pulmonary venous flow to the left side of the heart, and finally reduces stroke volume [8]. The opposite occurs during expiration. The respiratory modulation of stroke volume will directly influence the amplitude of OscP and then affect the BP measured using the automated oscillometric method. On the other hand, although the genesis of KorS is still a matter of debate, one longstanding hypothesis is that KorS is generated by the distension of the arterial wall caused by the changing transmural pressure gradient [9–12]. The respiratory modulation of stroke volume will directly influence the force deployed in opening the artery and then influence the blood flow sound, which will be reflected on the amplitude of the KorS signal and then influence the BP measured through the manual auscultatory method.

Some earlier studies have preliminarily investigated the effect of respiration on OscP and KorS recordings. Di Marco et al. [13] showed the respiratory modulation on KorS, but they did not quantitatively analyze the different respiratory modulation effects between normal and deep breathing. Zheng et al. [8] extended those studies and demonstrated the modulation effect of respiration on both OscP and KorS, but their study was performed under only static cuff pressures. There were significant differences between conditions of static cuff pressure and normal clinical BP measurement with deflating cuff pressure. As the cuff pressure during clinical BP measurement significantly influences the amplitudes of OscP and KorS, this may make the derived respiratory amplitude modulation effect on those two signals different from that observed with static cuff pressures. Therefore, studies are needed to explore the modulation effect of respiration on OscP and KorS during normal clinical BP measurement with deflating cuff pressures.

In order to make the investigation close to actual clinical conditions, the present study aimed to investigate the respiratory modulation effect on OscP and KorS during normal BP measurement. Two respiratory patterns [normal breathing and regular deep breathing] were used in this study to quantitatively assess: (1) the presence of amplitude modulation of respiration on OscP and KorS recordings; and (2) the differences in respiratory modulation on OscP and KorS between the two respiratory patterns.

## Methods

### Subjects and data acquisition

40 healthy subjects were enrolled in the study and their general information is summarized in Table 1. The study was carried out according to the Declaration of Helsinki (1989) of the World Medical Association, and was approved by Newcastle and North Tyneside NHS Research Ethics Committee. Informed and written consent was obtained from all subjects.

**Table 1:** General data information for the subjects studied

Subject information				
No. of subjects	40			
No. of male subjects	30			
No. of female subjects	10			

	Min	Max	Mean	SD
Age (years)	23	65	43	12
Height (cm)	152	192	173	9
Weight (kg)	50	105	75	11
Arm circumference (cm)	24	33	28	3
Resting SBP (mmHg)	94	140	115	13
Resting MAP (mmHg)	72	104	89	8
Resting DBP (mmHg)	61	90	76	7

Resting BP was collected from manual auscultatory measurement during standard cuff deflation

*MAP* mean arterial pressure

Manual SBP and DBP were measured by a trained operator according to the recommendations of the British and European Hypertension Societies [14]. The cuff was inflated to 200 mmHg and then deflated linearly at 2–3 mmHg/s. For each subject, six BP measurements were performed in total, with three repeats under two breathing patterns (normal and regular deep breathing with subjects breathing at their own comfortable rate). The order of the two breathing patterns was randomized between subjects. Between every two consecutive measurements, a resting period of appropriately 1 min was given to allow cardiovascular stabilization.

During linear cuff deflation, KorS was recorded by a piezo-electric microphone, with the bell-shaped stethoscope terminal connected to the microphone and placed on the antecubital fossa of the forearm. OscP was derived from the cuff pressure signals, while the reference respiration signal (Resp) was obtained by a chest magnetometer detecting chest wall movement [15]. All signals were digitally recorded at 2 kHz and 16 bits, and stored to a computer for offline processing.

### Signal processing

**Fig. 1:**
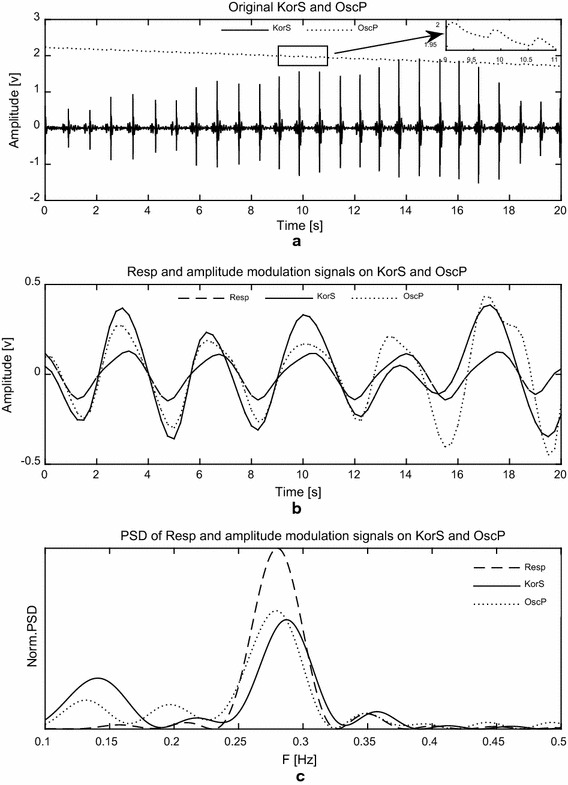
Signal processing procedure to analyze the amplitude modulation effect of respiration on OscP and KorS. **a** Original OscP (*dotted line*) and KorS (*solid line*) acquired during cuff deflation between SBP and DBP. The OscP signal between 9 and 11 s (marked with *rectangular*) is zoomed in and shown on the *top-right corner of panel*; **b** Reference Resp signal (*dashed line*) and amplitude modulation signals on OscP (*dotted line*) and KorS (*solid line*); and **c** normalized power spectral density of Resp (*dashed line*) and amplitude modulation signals on OscP (*dotted line*) and KorS (*solid line*)

Figure 1 shows the signal processing procedure to analyze the amplitude modulation effect of respiration on OscP and KorS during cuff deflation between SBP and DBP (a). On panel (b), reference Resp (from magnetometer) and the amplitude modulation signals on OscP and KorS are displayed to show the amplitude modulation effect of respiration on OscP and KorS. On panel (c) the normalized power spectral density of Resp and the amplitude modulation signals on OscP and KorS are displayed to show the respiratory amplitude modulation effect in the frequency domain. In the following sub-sections, each step of the signal processing procedure is described. Analysis was performed on anonymised data.

#### Reference respiration signal

Reference Resp signal was firstly down-sampled to 4 Hz (after low-pass filtering to prevent aliasing). This sampling rate satisfies the Nyquist condition, as the normal range of respiratory rates of healthy adults under resting conditions is between 0.1 and 0.5 Hz. Respiration depth was defined as the mean of peak-to-valley differences of Resp [8].

#### Amplitude modulation signals on OscP and KorS

The linearly deflating baseline cuff pressure was first removed to obtain OscP. The peak and valley of OscP and KorS pulses were then located. All pulse amplitudes of OscP and KorS were calculated as the amplitude difference between the peak and the valley. These amplitudes were resampled at 4 Hz by cubic spline interpolation to generate the amplitude modulation signals on OscP and KorS.

As shown in Fig. 1b, the cycles of Resp and amplitude modulation signals on OscP and KorS are nearly identical, indicating the amplitude modulation effect of respiration on OscP and KorS.

#### Amplitude modulation depth (AMD) between two breathing conditions

Over the time period between manual SBP and DBP, AMD was defined as the mean change of amplitude modulation signals normalized to mean amplitude of OscP and KorS signals. AMD was then used to investigate the difference in amplitude modulation of respiration on OscP and KorS between the two breathing patterns.

#### Respiratory frequencies from Resp and amplitude modulation signals on OscP and KorS

The normalized power spectral density of Resp and amplitude modulation signals on OscP and KorS were estimated by the Welch periodogram using a Hamming window (between 0.1 and 0.5 Hz and with frequency resolution of 0.001 Hz). Respiratory frequencies from Resp (f_R_), OscP (f_O_) and KorS (f_K_) were calculated as the peak frequency of the corresponding power spectral density. As shown in the example in Fig. 1c, the peak frequencies of the power spectral density of Resp and amplitude modulation signals on OscP and KorS are almost identical, indicating the amplitude modulation effect of respiration on OscP and KorS.

### Statistical analysis

One-way analysis of variance (ANOVA) was used to analyze the repeatability between the three repeated measurements. The two-tail t test (with a significance level of α = 0.05) was used to assess BP, respiratory frequency, respiratory depth and AMD between the two respiratory patterns. Bland–Altman analysis with limits of agreement was used to investigate the agreement between f_R_ and f_O_ or f_K_ [16].

## Results

### Repeatability between measurements

There was no statistically significant difference (all *p* > 0.05) for all the parameters derived in this study for the three repeat recordings, indicating that subjects kept relatively still and breathed at a relatively stable rate for both conditions. This was important for enabling changes to be quantified for the different measurements.

### Blood pressure changes with regular deep breathing

Table 2 presents the BPs (SBP, MAP and DBP) measured manually under normal and regular deep breathing conditions. With deep breathing, there were statistically significant decreases for all BPs (SBP, MAP and DBP) (all *p* < 0.001), confirming that regular deep breathing influenced BP.

**Table 2:** BP parameters in normal and deep breathing conditions

Parameter	Normal breathing	Deep breathing	*p* value
SBP (mmHg)	115 ± 2	111 ± 1	<0.001
MAP (mmHg)	89 ± 1	85 ± 1	<0.001
DBP (mmHg)	76 ± 1	72 ± 1	<0.001

Data are presented as mean ± SE (standard error)

### Reference respiratory parameters under the two respiratory patterns

As shown in Table 3, regular deep breathing significantly (all *p* < 0.001) decreased respiratory rate, and increased respiratory depth in comparison with the normal breathing condition.

**Table 3:** Respiratory parameters in normal and deep breathing conditions

Parameter	Normal breathing	Deep breathing	*p* value
f_R_ (Hz)	0.25 ± 0.01	0.19 ± 0.01	<0.001
Respiratory depth (arbitrary unit)	0.16 ± 0.02	0.47 ± 0.06	<0.001

Data are presented as mean ± SE

### Amplitude modulation depth difference under normal and deep breathing conditions

As shown in Table 4, AMD increased significantly (both *p* < 0.05) from 14 to 40 % for OscP and KorS respectively under normal breathing condition to 16 and 49 % with deeper breathing, indicating a significantly stronger amplitude modulation effect on both OscP and KorS signals with deep breathing.

**Table 4:** Amplitude modulation depth of OscP and KorS in normal and deep breathing conditions

Signal type	Normal breathing	Deep breathing	*p* value
OscP (%)	14 ± 1	16 ± 1	<0.05
KorS (%)	40 ± 3	49 ± 4	<0.05

Data are presented as mean ± SE

### Derived respiratory frequency difference under normal and deep breathing conditions

**Fig. 2:**
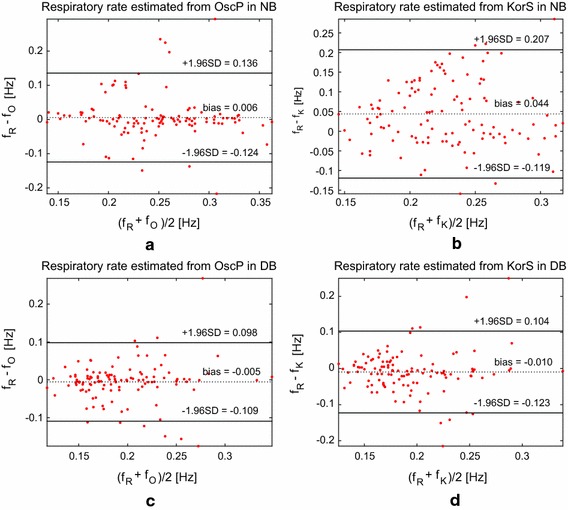
Bland–Altman difference plot of respiratory frequency estimation from OscP and KorS in normal and deep breathing conditions. *Dotted black line* indicates bias and *solid black lines* indicate 1.96-SD limits of agreement

**Fig. 3:**
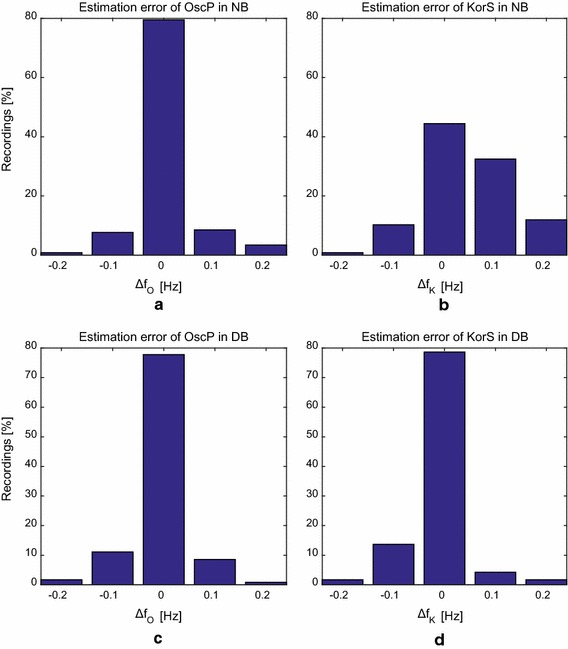
Histogram of respiratory frequency estimation error from OscP and KorS under normal and deep breathing conditions. Frequency bin width is 0.1 Hz

Figures 2 and 3 show respiratory frequency estimation errors for OscP and KorS in comparison with f_R_ under both normal and deep breathing conditions. In comparison with f_R_, over 60 % measurements of f_O_ and f_K_ agreed with f_R_ within 0.05 Hz or less. This was increased to nearly 80 % with deeper breathing. There was no statistically significant difference between the reference respiration frequency and these derived from the OscP and KorS signals (both *p* > 0.05) during deep breathing, and for the OscP signal during normal breathing (*p* > 0.05). As given in Table 5, the minimum frequency difference was observed from OscP in deep breathing with the smallest limit of agreement of [−0.109 to 0.098 Hz].

**Table 5:** Comparison of f_R_ with f_O_ and f_K_

Parameter	Mean bias (Hz)	Limit of agreement (Hz)
Normal breathing		
f_O_	0.006	[−0.124,0.136]
f_K_	0.044	[−0.119,0.207]
Deep breathing		
f_O_	−0.005	[−0.109,0.098]
f_K_	−0.010	[−0.123,0.104]

## Discussion

In this study, normal and regular deep breathing were adopted at each subject’s comfortable respiratory rate to study the influence of respiration on OscP and KorS recordings. As expected and agreed with published studies [13, 17], regular deep breathing significantly decreased respiratory rate, and increased respiratory depth in comparison with the normal breathing condition. The statistically significant difference for respiratory parameters between regular deep breathing and normal breathing was a precondition for studying the different modulation effects of respiration on OscP and KorS between breathing conditions.

Our study showed that the amplitude modulation depths of respiration on OscP and KorS were significantly higher under deep breathing condition. This might be attributed to the significantly higher respiratory depth with deep breathing which increases the amplitude modulation effect of respiration on OscP and KorS. This result was in accordance with the findings of Saul et al. [18], where a direct mechanical coupling between respiration and the vasculature has been shown. The results also agreed with what we have reported on the respiratory effect on OscP recorded under static pressures [8], but the difference in amplitude modulation effect on KorS had not been observed in the previous study. This could be caused by the signals recorded from different measurement conditions. The KorS signal in our previous study was recorded from static pressures between 0.2 and 0.3 Hz, while the current one was from deflating cuff pressures between 0.19 and 0.25 Hz. In the future, it would be worth investigating the relationship between respiratory rate and the modulation effect.

This study also extended the study of Di Marco et al. [13] and Zheng et al. [8], and showed the amplitude modulation effect of respiration on OscP and KorS during normal clinical BP measurement. Although the amplitudes of OscP and KorS change with deflating cuff pressure, a good agreement between f_R_ and f_O_ or f_K_ has been observed. The derived respiration frequency from OscP and KorS signals during deep breathing, and from the OscP signal during normal breathing had no statistically significant difference from the reference frequency, indicating the respiratory modulation effect on OscP and KorS during normal clinical BP measurement. This also reveals the potential to derive respiration rate from OscP during BP measurement.

The agreement between the reference respiratory frequency and that derived from KorS under normal breathing condition was not as good as that under the deep breathing condition. This could also be caused by lower amplitude modulation depth under normal breathing condition, which makes the respiratory amplitude modulation signal on KorS more sensitive to noise interference.

Note that a limitation of this work is that only healthy subjects with normal blood pressure were recruited to participate in this study. There may be differences in the magnitude of the effect of respiration on BP measurements in patients with cardiovascular disease due to factors such as differences in central arterial stiffness. A further clinical study is warranted to compare the amplitude modulation effect of respiration on OscP and KorS between patients with known cardiovascular disease (e.g., hypotensive and hypertensive) and age-matched healthy subjects.

## Conclusions

In conclusion, we have provided scientific evidence for (1) the presence of an amplitude modulation effect of respiration on OscP and KorS during normal clinical BP measurement, and (2) the significantly stronger amplitude modulation effect of respiration on OscP and KorS for regular deep breathing than for the normal breathing condition.

## Abbreviations

AMD: amplitude modulation depth
ANOVA: analysis of variance
BP: blood pressure
DBP: diastolic blood pressure
f_K_: frequency of the amplitude modulation signal on Korotkoff sound
f_O_: frequency of the amplitude modulation signal on oscillometric cuff pressure pulses
f_R_: frequency of respiration signal
JAMA: Journal of the American Medical Association
KorS: Korotkoff sound
MAP: mean arterial pressure
OscP: oscillometric cuff pressure pulses
Resp: respiration signal
SBP: systolic blood pressure
Δf_K_: f_R_ − f_K_
Δf_O_: f_R_ − f_O_
